# Persistent tumor cells in bone marrow of non-metastatic breast cancer patients after primary surgery are associated with inferior outcome

**DOI:** 10.1186/1471-2407-12-190

**Published:** 2012-05-28

**Authors:** Kjersti Tjensvoll, Satu Oltedal, Reino Heikkilä, Jan Terje Kvaløy, Bjørnar Gilje, James M Reuben, Rune Smaaland, Oddmund Nordgård

**Affiliations:** 1 Department of Haematology and Oncology, Stavanger University Hospital, N-4068, Stavanger, Norway; 2 Department of Mathematics and Natural Sciences, University of Stavanger, N-4036, Stavanger, Norway; 3 Division of Research and Human Resources, Stavanger University Hospital, N-4068, Stavanger, Norway; 4 Department of Hematopathology, The University of Texas M. D. Anderson Cancer Center, Houston, TX, 77030, USA; 5 Laboratory for Molecular Biology, Department of Haematology and Oncology, Stavanger University Hospital, N-4068, Stavanger, Norway; 6 Present address: Roche Norge AS, 0915, Oslo, Norway

**Keywords:** Breast cancer, Minimal residual disease, Multimarker real-time PCR, Bone marrow, DTC, prognosis

## Abstract

**Background:**

To investigate the prognostic significance of disseminated tumor cells (DTCs) in bone marrow (BM) from non-metastatic breast cancer patients before and after surgery.

**Methods:**

Patients with non-metastatic breast cancer were consecutively recruited to this project during the years 1998–2000. Real-time RT-PCR quantification of a DTC multimarker panel consisting of cytokeratin 19, mammaglobin A and TWIST1 mRNA was performed in BM samples obtained from 154 patients three weeks (BM2) and/or six months after surgery (BM3). The results were compared to previously published data from pre-operative BM analyses for the same patients.

**Results:**

DTCs were identified in post-operative BM samples (BM2 and/or BM3) from 23 (15%) of the 154 patients investigated. During a median follow-up of 98 months, 10 (44%) of these patients experienced systemic relapse as compared to 16 (12%) of 131 DTC-negative patients. Kaplan-Meier estimates of systemic recurrence-free- and breast-cancer specific survival demonstrated significantly shorter survival for patients with persistent DTCs in BM after surgery (p≤0.001). By multivariate Cox regression analyses, persistent DTCs after surgery was an independent predictor of both systemic recurrence-free- (HR = 5.4, *p* < 0.001) and breast-cancer specific survival (HR = 5.3, *p* < 0.001). Furthermore, the prognostic value of DTCs in BM was similar for pre- and post surgery samples. However, patients with DTCs both before and after surgery (BM1 and BM2/3) had a particularly poor prognosis (systemic recurrence-free survival: HR = 7.2, *p* < 0.0001 and breast-cancer specific survival: HR = 8.0, *p* < 0.0001).

**Conclusions:**

Detection of persistent DTCs in BM samples obtained after surgery identified non-metastatic breast cancer patients at high risk for systemic relapse, and with reduced breast-cancer specific survival. Furthermore, patients with positive DTC status both before and after surgery had a particularly poor prognosis.

## Background

There is considerable evidence that detection of disseminated tumor cells (DTCs) in pre-operative bone marrow (BM) samples from non-metastatic breast cancer patients identifies a patient population at high risk for disease recurrence [1-5]. DTCs have also been found in the BM after surgery, both before and after adjuvant treatment [6-12]. The shedding of cells observed from primary tumors should be expected to end after removal of the tumor by surgery. Thus, DTCs detected after radical surgery in patients without evidence of distant metastases must originate from occult metastases or be able to persist in the BM after surgery. DTCs which persist post-operatively after surgery, and even after completion of adjuvant treatment, may be enriched for tumor cells with better capability to survive, and also grow, in the secondary site. Accordingly, it has been demonstrated that a large fraction of the breast cancer DTCs has a stem cell-like phenotype [13,14], which may cause resistance to conventional chemotherapeutic drugs [15]. Thus, both a prognostic and a predictive value of BM DTC detection after surgery seem likely. Furthermore, one may hypothesize that repeated BM sampling in order to detect DTCs, in particular following administration of adjuvant treatment, may improve the prediction of disease recurrence and the selection of patients who might benefit from secondary or intensified adjuvant treatment.

There is limited evidence of the clinical usefulness of the suggested repeated BM sampling. Two studies, both using immunocytochemical detection methods, have reported the results of repeated BM sampling performed in women who were recurrence-free 2–3 years after primary diagnosis. The presence of DTCs in this group of patients significantly predicted shorter distant disease-free survival, but the prognostic impact seemed similar to that obtained from pre-operative analyses [6,7]. To select patients who would benefit by secondary adjuvant treatment, repeated BM sampling at an earlier time might be preferable. One such study has been reported by Daskalaki *et al*. (2009), with samples collected before and after adjuvant chemotherapy [10]. In contrast to the two prior studies, this group used real-time RT-PCR quantification of cytokeratin 19 (CK19) transcripts to detect DTCs. They observed a survival difference according to DTC status both before and after chemotherapy, however, the difference between the DTC positive and negative groups was statistically significant only for the BM samples obtained before chemotherapy [10].

In the present study, we have used real-time RT-PCR quantification of a multimarker (MM) mRNA panel to detect DTCs in BM. The use of our MM mRNA panel is expected to result in high sensitivity since the markers may be differentially expressed in breast cancer cells. Previously we have demonstrated, using our MM mRNA panel, that detection of DTCs in pre-operative BM samples predicts clinical outcome in non-metastatic breast cancer patients [16]. The present study is the first reporting repeated post-operative BM samples from non-metastatic breast cancer patients assessed by a MM quantitative RT-PCR assay for DTC detection. The BM samples were obtained three weeks and six months after surgery from 154 patients. Having 98 months (>8 years) median follow-up data for the patients, we have evaluated the prognostic significance of persistent DTCs in BM after surgery, and compared the prognostic and predictive information associated with the different sampling time points.

## Methods

This study is reported according to the recommendations for tumor marker prognostic studies [17].

### Patient cohort

Initially 234 patients (median age 56 years, range 25–86 years) with non-metastatic breast cancer (M0) were consecutively recruited to this project during the period 1998–2000. However, forty-three patients were excluded from the study; 25 patients with either ductal carcinoma *in situ* (DCIS) or lobular carcinoma *in situ* (LCIS), 7 patients with benign lesions, one patient with primary metastatic disease and 10 patients with missing BM samples. The prognostic impact of DTCs in BM samples obtained prior to surgery (BM1) has previously been evaluated in the remaining 191 patients [16,18,19].

In the present study, we have analyzed additional BM samples (20 mL in heparin anticoagulant) that were obtained by unilateral aspiration from the posterior iliac crest under local anesthesia three weeks (denoted BM2), and six months (denoted BM3) after primary surgery. However, after surgery only 144 of the 191 included patients consented to having a second BM aspiration (BM2), while 109 patients agreed to undergo a third BM aspiration (BM3). In total, BM2 and/or BM3 aspirates were obtained from 154 patients (for more details see Table 1), and 99 patients allowed aspirations at all three time points.

**Table 1:** Comparison of the clinicopathological parameters of the patients according to DTC status in bone marrow after primary surgery

**Variable**	**No. of patients n = 154**	**Post-operatively DTC status**		**P-values**
		**Positive n = 23**	**Negative n = 131**	
**Age**				**0.013**
<=55 years	78	6 (8)	72 (92)	
>55 years	76	17 (22)	59 (78)	
**Lymph node status**				0.223
pN0	110	14 (13)	96 (87)	
pN1-2	44	9 (20)	35 (80)	
**Tumor size**				0.469
pT1	105	14 (13)	91 (87)	
pT2-4	49	9 (18)	40 (82)	
**Tumor grade**				0.803
1	55	8 (14)	47 (86)	
2	61	10 (16)	51 (84)	
3	35	4 (11)	31 (89)	
Unknown	3			
**Estrogen receptor status**				0.365
ER positive	127	17 (13)	110 (87)	
ER negative	25	5 (20)	20 (80)	
Unknown	2			
**Progesterone receptor status**				0.105
PgR positive	76	15 (20)	61 (80)	
PgR negative	75	7 (9)	68 (91)	
Unknown	3			
**Histological type**				0.182
Ductal	121	15 (12)	106 (88)	
Lobular	15	3 (20)	12 (80)	
Mixed ductal/lobular	5	1 (20)	4 (80)	
Other	13	4 (31)	9 (69)	
**Chemotherapy**				0.798
Therapy	41	5 (12)	36 (88)	
No therapy	113	18 (16)	95 (84)	
**Endocrine therapy**				0.336
Therapy	47	9 (18)	38 (81)	
No therapy	107	14 (13)	93 (87)	

Numbers in brackets represent the number of patients in percent.

The patients were treated according to the Norwegian National guidelines at that time, and the treatment and clinical follow-up of the patients were done systematically as previously described [18]. In detail, 30 of the 99 patients received adjuvant chemotherapy and 34 patients received adjuvant endocrine therapy. Due to some overlap between the treatment groups, 44 of 99 patients received adjuvant endocrine- and/or adjuvant chemotherapy, whereas 55 did not receive any adjuvant treatment.

Follow-up data for all patients were collected from the hospital records and from their primary physician’s records. The control program for the patients was according to the routines of the institution, with one to two visits per year, depending on patient age, stage, breast conserving treatment versus mastectomy, time from primary treatment and method of diagnosis (screening-detected vs. not). Blood tests were performed 1–2 times per year, in addition to mammography examination once a year. Information on time of death was obtained from the Hospital records, through an automatic update from the National Registry in Norway. The cause of death was determined from the medical files at the hospital, or by information from the patients’ primary physicians [19]. The end of the follow-up period was October 2008, and the median follow-up time was 98 months (range 1–127 months). The project was approved by the Regional Committees for Medical and Health Research Ethics, and written informed consent was obtained from all participating patients. Single BM aspirates obtained from 26 healthy women constituted the control group.

### RNA isolation and cDNA synthesis

BM lysates were prepared from buffy coat as previously described [20]. Total RNA was isolated, and A260/A280 ratios measured (range 1.9–2.0). For a subset of the samples the RNA quality was also measured on the Agilent 2100 Bioanalyzer (range RIN = 8.2–9.3). Afterwards, the RNA was treated with DNase I and reverse transcribed to cDNA [18]. All cDNA samples were stored at −80°C.

### qRT-PCR

Amplification of the MM panel consisting of CK19 (NM_002276), hMAM (U33147) and TWIST1 (NM_000474) mRNA was performed as described previously [16,18,19]. However, in order to increase the sensitivity for hMAM mRNA detection, we increased the cDNA content from 20 to 50 ng, and reduced the primer concentration to 0.3 μM compared to that previously described by Tjensvoll *et al*. (2009) [19]. Quantification of the three mRNA markers was performed, blinded for the identity and clinical outcome of the patients, in a LightCycler 480 (Roche Applied Science) instrument. The breakpoint cluster region (BCR, NM_004327) was used as a reference gene. CK19 and TWIST1 were analysed in duplicates, while hMAM was analysed in triplicates.

### Relative mRNA quantification

mRNA concentrations, based on mean crossing point (CP) values, were normalized against BCR mRNA level and expressed relative to a calibrator sample as previously described [16,19]. A cut-off value was determined for each marker based on the highest mRNA level in BM samples of the normal control group (n = 26). The relative hMAM and TWIST1 mRNA concentrations have been determined previously in the BM samples from this control group of healthy women [16,19], whereas the CK19 mRNA concentration was measured in the present study. The highest relative CK19 mRNA level determined in the normal control BMs was 7.40×10^−4^, and this was used as a threshold for normal mRNA level in the further analyses. BM samples were considered as positive for DTCs when positive for at least one of the mRNA markers (i.e. CK19, hMAM or TWIST1).

### Statistical analyses

The statistical analyses were performed in SPSS version 17.0 (http://www.spss.com) with a two-sided p-value ≤0.05 considered as statistically significant. P-values were not corrected for multiple testing, and missing data were excluded from the analyses. Relations between the multimarker BM expression and various clinicopathological parameters were tested by Fisher’s exact test. Kaplan-Meier estimates of clinical outcome were determined for the time intervals from primary surgery to A) systemic (distant) recurrence of the disease (systemic recurrence-free survival) and B) death related to progression of breast cancer (breast-cancer-specific survival).

Cox univariate and multivariate survival regression was used to evaluate the effects of BM DTC status, lymph node (LN) status, tumor size, tumor grade, age, estrogen receptor (ER) status, progesterone receptor (PR) status, adjuvant chemotherapy and endocrine therapy on systemic-recurrence-free survival and breast-cancer specific survival (see [16,19]). The multivariate analyses were performed using both forward and backward stepwise selection of covariates. The effect of each variable in these models was assessed by the Wald test and described by the hazard ratio (HR), with a 95% confidence interval.

## Results

### Detection of persistent DTCs in BM samples obtained after primary surgery

We have previously demonstrated by a MM quantitative RT-PCR panel that detection of DTCs in pre-operative BM samples (BM1) predicts clinical outcome in non-metastatic breast cancer patients [16]. In order to evaluate the presence of persistent DTCs as a marker for poor outcome, BM samples were obtained three weeks (BM2, n = 144) and/or six months (BM3, n = 109) after primary surgery from 154 of the breast cancer patients (Table 1) previously analysed for pre-operative (BM1) DTC status. The BM sample was considered to contain DTCs (positive DTC status) if at least one of the surrogate mRNA markers CK19, hMAM and TWIST1 had levels above the established threshold values [16,19]. Of the 154 analysed patients with early breast cancer, 23 (15%) patients were DTC-positive in at least one of the post-operative BM samples (Table 2). Potential associations between clinicopathological parameters and DTC status in BM after surgery (at three weeks and/or six months) are shown in Table 1. Furthermore, eight patients were DTC positive both pre- (BM1) and post-operatively (BM2 and/or BM3), whereas 1/99 patients who had BM aspirates drawn at all three time points was DTC positive in all three samples.

**Table 2:** The number of positive bone marrow (BM) samples shown separately for the three mRNA markers mammaglobin A (hMAM), cytokeratin 19 (CK19) and TWIST1, as well as in combination by construction of a multimarker panel

** *BM samples* **	** *No. of * *patients* **	** *Missing* *data* **	** *Marker expression* **			** *Multimarker* ** positive*
			** *hMAM* **	** *CK19* **	** *TWIST1* **	
BM1	154	0	4 (3)	14 (8)	5 (2)	21 (11)
BM2	144	10	6 (5)	5 (2)	9 (4)	17 (8)
BM3	109	45	6 (3)	1 (1)	2 (1)	7 (3)
BM2 and/or BM3	154	0	11 (7)	5 (2)	11 (5)	23 (10)

*BM1* drawn prior to surgery; *BM2* drawn three weeks after primary surgery; *BM3* drawn six months after primary surgery.

The number of systemic relapse is shown in parentheses.

*Positive for at least one of the markers hMAM, CK19 and TWIST1.

Since not all of the patients who provided a BM aspiration prior to surgery agreed to provide BM aspirations three weeks (BM2) and/or six months after surgery (BM3), we tested by Fisher’s exact test if there were any biases between the different sample groups included in this study. No significant differences were found between the 45 patients only providing BM2, and the 99 patients who provided both BM2 and BM3 aspirations. However, there was a trend (*p* = 0.12) towards a higher frequency of N0 patients with lower differentiation grade in the BM2 sampling group.

In the BM samples obtained after primary surgery the three mRNA markers were complementary, as only four of 154 patients had elevated levels of more than one marker (Table 2). Furthermore, the relative contribution of the CK19 marker to the total number of DTC positive samples declined in the post-operative BM samples, as compared with the contribution of this marker detected prior to surgery (Table 2).

### Prognostic significance of persistent DTCs in BM after primary surgery

**Figure 1:**
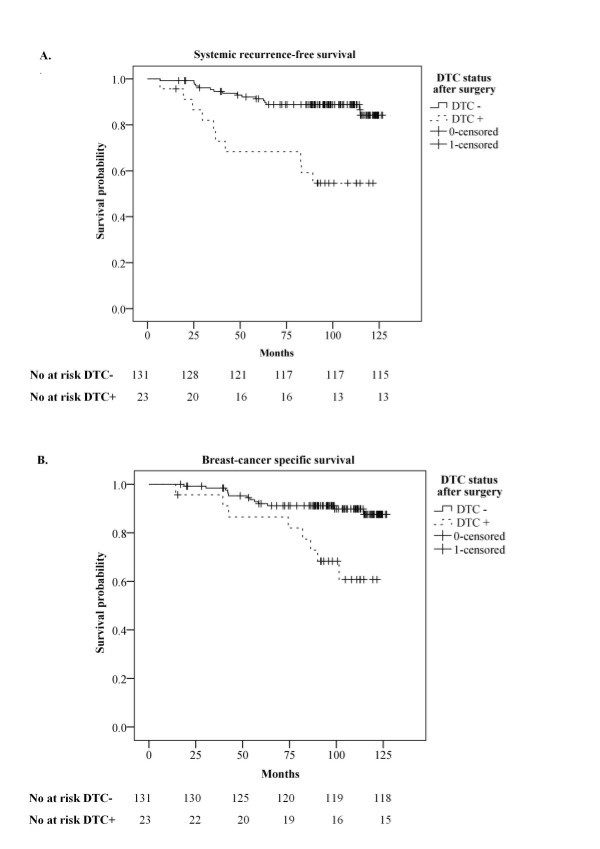
**Kaplan Meier estimates according to the presence of persistent disseminated tumor cells in bone marrow after surgery.** Kaplan-Meier estimates of systemic recurrence-free survival **(A)**, and breast-cancer specific survival **(B)** according to positive (n = 23) and negative (n = 131) persistent disseminated tumor cell (DTC) status in bone marrow as detected three weeks and/or six months after surgery. P-values were calculated by the log-rank test. The numbers of patients at risk are indicated below each plot.

During a median follow-up of 98 months, 10 out of the 23 (44%) patients with persistent DTCs detected in at least one of the two post-operative BM samples experienced systemic relapse (Table 2), compared with 16 of 131 (12%) patients with negative DTC status in post-operative BM. Eight of the 10 (80%) patients with recurrent disease have subsequently died from breast cancer. Kaplan-Meier estimates of systemic recurrence-free survival and breast-cancer specific survival both showed that the DTC status in BM samples obtained after primary surgery was a strong prognostic factor (Figure 1). A significantly shorter overall- (*p* = 0.006) and recurrence-free survival (*p* < 0.001) was also demonstrated (data not shown). Moreover, when we stratified the Kaplan-Meier analyses of systemic recurrence-free survival according to LN status, the post-operative DTC status was demonstrated to be a strong prognostic factor in both the LN-negative (*p* = 0.007) and the LN-positive patients (*p* = 0.010, curves not presented).

Uni- and multivariate Cox regressions were performed to evaluate the prognostic impact of persistent DTCs in BM of non-metastatic breast cancer patients. In the univariate analyses DTC status at all the three sampling time points, LN status, tumor grade and ER status were significant predictors of both systemic recurrence-free survival and breast-cancer specific survival (Table 3). The multivariate analyses showed that the presence of DTCs in post-operative BM samples, as detected by our MM quantitative RT-PCR assay, was a strong independent prognostic factor (Table 4) together with lymph node status and tumor grade (data not presented). This was demonstrated by both forward and backward stepwise selection of variables.

**Table 3:** Univariate Cox regression analyses of systemic-recurrence-free survival and breast-cancer specific survival

**BM samples**	**Parameter**	**Hazard ratio**	**95% CI**	**P-values**
	**Systemic recurrence-free survival**			
BM1	DTC status (pos. vs. neg.)	5.944	2.713–13.022	**<0.001**
BM2*	DTC status (pos. vs. neg.)	5.013	2.133–11.782	**<0.001**
BM3*	DTC status (pos. vs. neg.)	3.680	1.062–12.744	**0.040**
BM2 and/or BM3	DTC status (pos. vs. neg.)	4.523	2.044–10.008	**<0.001**
BM1 and BM2/3	DTC status (pos. vs. neg.)	9.948	4.103–24.122	**<0.001**
	Lymph node status (pN > 0 vs. pN0)	3.995	1.833–8.708	**<0.001**
	Tumor size (pT3 and pT4 vs. pT2 vs. pT1)	1.523	0.691–3.358	0.297
	Tumor grade (3 vs. 2 vs. 1)	2.015	1.212–3.352	**0.007**
	Age (>55 or not)	1.044	0.484–2.252	0.913
	ER (positive vs. negative)	0.322	0.143–0.723	**0.006**
	PgR (positive vs. negative)	0.735	0.337–1.605	0.440
	Adjuvant chemotherapy (received or not)	1.603	0.713–3.601	0.253
	Adjuvant endocrine therapy (received or not)	1.814	0.832–3.951	0.134
	**Breast-cancer specific survival**			
BM1	DTC status (pos. vs. neg.)	5.408	2.271–12.880	**<0.001**
BM2*	DTC status (pos. vs. neg.)	3.880	1.470–10.238	**0.006**
BM3*	DTC status (pos. vs. neg.)	4.626	1.286–16.635	**0.019**
BM2 and/or BM3	DTC status (pos. vs. neg.)	3.851	1.592–9.316	**0.003**
BM1 and BM2/3	DTC status (pos. vs. neg.)	9.205	3.526–24.031	**<0.001**
	Lymph node status (pN > 0 vs. pN0)	4.694	1.944–11.344	**0.001**
	Tumor size (pT3 and pT4 vs. pT2 vs. pT1)	1.788	0.753–4.247	0.188
	Tumor grade (3 vs. 2 vs. 1)	3.288	1.743–6.201	**<0.001**
	Age (>55 or not)	1.384	0.583–3.284	0.461
	ER (positive vs. negative)	0.217	0.091–0.516	**0.001**
	PgR (positive vs. negative)	0.729	0.306–1.733	0.474
	Adjuvant chemotherapy (received or not)	1.545	0.623–3.833	0.348
	Adjuvant endocrine therapy (received or not)	1.820	0.766–4.324	0.175

*BM1* drawn prior to surgery, *BM2* drawn three weeks after surgery, *BM3* drawn six months after surgery;*BM* bone marrow; *DTC* disseminated tumor cell; *ER* Estrogen receptor; *PgR* Progesterone receptor; *BM2/3* BM2 and/or BM3.

*only patients with this sample available were included in the analysis.

**Table 4:** Multivariate Cox regression analyses of systemic recurrence-free survival, and breast-cancer specific survival according to DTC detection in BM samples drawn at different time points from non-metastatic breast cancer patients

**BM samples**	**DTC-positive patients**	**Systemic recurrence-free survival**		**Breast-cancer specific survival**	
		**Hazard ratio**	**P-values**	**Hazard ratio**	**P-values**
BM1	16%	6.420	<0.001	7.081	<0.001
BM2	12%	5.793	<0.001	6.455	0.001
BM3	6%	6.841	0.004	4.888	0.022
BM2 and/or BM3	15%	5.397	<0.001	5.303	<0.001
BM1 and BM2/3	5%	7.188	<0.001	8.018	<0.001

*BM1* drawn prior to surgery; *BM2* drawn three weeks after surgery; *BM3* drawn six months after surgery.

The different BM samples were included in the models in separate regression experiments. Only results from backward stepwise selection of variables are presented.

### Comparison of the prognostic significance of DTCs in BM samples obtained at different time points

**Figure 2:**
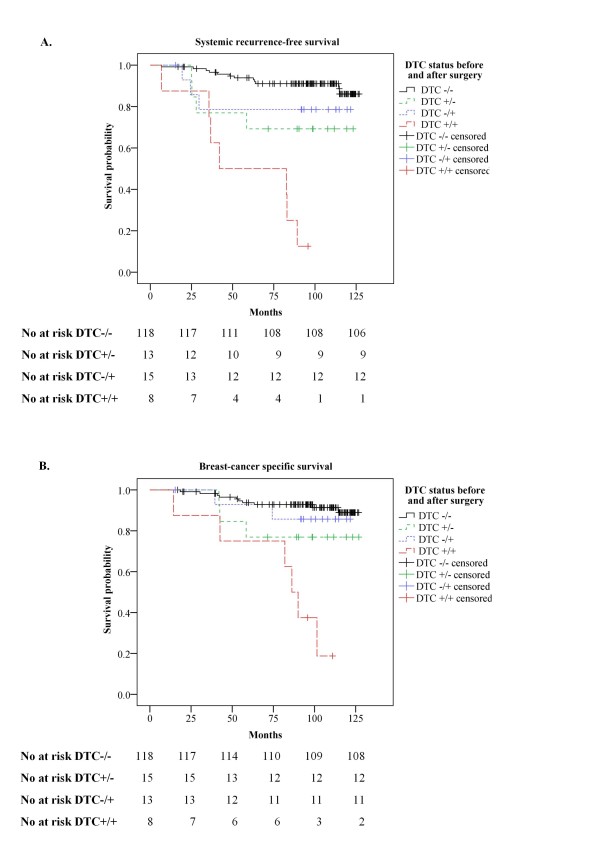
**Kaplan Meier estimates according to the presence of disseminated tumor cells in bone marrow before and after surgery.** Kaplan-Meier estimates of systemic recurrence-free survival **(A)**, and breast-cancer specific survival **(B)** according to the presence of disseminated tumor cells (DTCs) in bone marrow (BM) only before surgery (DTC +/−, n = 13), only after surgery (DTC −/+, n = 15), both before and after surgery (DTC +/+, n = 8) and no presence of DTCs in BM (DTC −/−, n = 118). P-values were calculated by the log-rank test. The numbers of patients at risk are indicated below each plot.

We compared the prognostic significance of DTC detection in BM samples obtained before (BM1), three weeks (BM2) and six months (BM3) after primary surgery by three separate multivariate Cox regressions, also including other prognostic factors, and found only small differences in the hazard ratios between the three time points (Table 4). However, the number of DTC-positive patients was substantially higher for the BM obtained before (BM1) as well as three weeks (BM2) after surgery (Table 4). Moreover, as suggested by the univariate Cox regression analysis, the combination of both pre- and post-operative positive DTC status remained a particularly strong prognostic factor in the multivariate analysis (Table 4). Kaplan-Meier survival analyses demonstrated that patients with positive DTC status both before and after primary surgery had an estimated 8-year systemic recurrence-free survival and breast-cancer specific survival below 20% (Figure 2).

## Discussion

We have previously demonstrated that the presence of DTCs, in terms of MM mRNA levels above the established cut-off values, in BM samples obtained prior to breast cancer surgery provide significant prognostic information (HR = 3.59, *p* = 0.001 for systemic-recurrence-free survival) [16]. In the present study, we have shown that DTCs in BM samples obtained three weeks and/or six months after surgery provide similar prognostic information as the DTC status in pre-operative BM, with hazard ratios in the range 5.8–6.8 (Table 4). These results were somewhat unexpected since the presumptive passive shedding of cells from primary tumors gives reason to believe that a higher proportion of clinically insignificant DTCs would be present in the BM before surgery. In this respect, BM sampling after surgery was expected to give more significant prognostic information. The results of the present study confirm the prognostic significance of DTCs after surgery as detected by our real-time RT-PCR assay. Most likely, the clinical importance of persistent DTCs in BM is even stronger than observed, as some of the included patients only agreed to one of the post-operative BM aspirations. Our results do not, however, suggest that patients with DTCs detected post-operatively have a prognosis that is inferior to patients with DTCs detected pre-operatively. Thus, a selection of DTCs with a higher capability of establishing clinical overt metastases after removal of the primary tumor, or even after six months of adjuvant treatment, is not supported. This finding is consistent with the report by Daskalaki *et al*. (2009), in which the estimated prognostic effect of DTCs in BM samples collected shortly after adjuvant chemotherapy was similar to the effect in BM samples collected post-operatively, prior to chemotherapy [10]. Similarly, the impact of DTCs in BM samples obtained 2–3 years after diagnosis seemed to have a similar magnitude as that observed pre-operatively [2,5-7].

The results in the present study do, however, suggest that a positive pre-operative DTC status, if confirmed in a second BM sample collected three weeks or six months post-operatively (double positive), predicts a particularly poor prognosis. The estimated 8-year systemic recurrence-free survival in this group of patients was in fact <20% (Figure 2A), as compared to >50% in the group defined as DTC positive based on post-operative BM samples alone (Figure 1A). A similar trend was reported by Wiedswang *et al.* (2004), although it was not as striking as in our study. In their study, the estimated 5-year distant disease-free survival was <70% for the patients with DTC-positive BM both at diagnosis and after three years, as compared to ~80% for the whole group of patients who were DTC positive only in the second BM sample [6]. However, the fact that they obtained the follow-up BM samples three years after surgery, and thus excluded the patients with recurrence during this 3-year interval, must be considered when comparing their estimated survival rates with ours. Our group of patients, with DTC positive BM both before and after surgery, included both LN-positive and LN-negative (N0) patients, the tumor size varying from T1-T4 with grade 1–3. Hence, these patients constituted a very heterogeneous group not necessarily destined to experience an unfavorable clinical outcome based on conventional prognostic factors.

It would be interesting to investigate whether DTC detection after adjuvant therapy could be a surrogate marker for evaluation of treatment efficiency. However, due to small patient numbers in the subgroups receiving adjuvant treatment it was not possible to conclude on the potential for monitoring or prediction of adjuvant treatment efficiency in this study. This important aspect should be addressed in new studies.

Discrepancies between the methods used to detect DTCs could make it difficult to compare studies using different methodologies. Immunocytochemistry has the advantages of being able to characterize cell size, cell shape and atypical enlargement of nucleus which may occur in malignant cells. However, due to the absence of tumor-specific targets, monoclonal antibodies against various epithelium-specific antigens, like the cytokeratins, are mostly used. In comparison, molecular methods are highly sensitive and may detect DTCs based on their expression of tumor-specific markers. One disadvantage is nevertheless that the cells cannot be morphologically characterized by the use of molecular methods. However, molecular profiling of breast cancer cells may be used to characterize and classify the tumor cells according to their protein, DNA or mRNA pattern.

In the present study we demonstrate that 15% of the patients with non-metastatic breast cancer have DTCs detected in BM after primary surgery (Table 4). Although the use of different methodologies complicates a direct comparison between the studies**,** our results seem to correspond with Wiedswang *et al*. (2004) also showing that 15% of the non-metastatic breast cancer patients had detectable DTCs in BM at a median 66 months from diagnosis [6]. Janni *et al*. (2005) detected DTCs in 13% of the patients after surgery [7]. An European pooled analysis involving 676 breast cancer patients also corroborates our findings; 15.5% of the breast cancer patients having DTCs detected in BM after surgery, and this was found to be an independent predictor of subsequent reduced breast-cancer specific survival [12]. The DTC frequency numbers published by Daskalaki *et al*. (2009) using real-time RT-PCR for detection of CK19 mRNA-positive DTCs is, however, in contrast to these results. In their study, 58% of the non-metastatic breast cancer patients had BM DTCs detected after surgery, prior to chemotherapy, and 51% after chemotherapy [10]. The low recurrence rate among the DTC-positive patients in their study suggests, however, that their assay is less specific with regard to clinical relevance. This may be partly due to a lower threshold for test positivity, as they have, as opposed to us, not used the highest determined normal BM level of the marker as a threshold [10].

We observed that the contribution of CK19 to DTC detection, relative to hMAM and TWIST1, was lower after surgery than prior to surgery (Table 2). This observation supports the hypothesis that the DTC population after surgery may be enriched for tumor cells undergoing EMT (epithelial to mesenchymal transition), being able to persist in the BM after primary surgery, as decreased expression of cytokeratins is associated with the EMT process [21]. A reduction in the number of CK19-positive patients after surgery has also been reported previously [9]. These observations also contrast somewhat with Daskalaki *et al*. (2009) regarding the high fraction of patients with CK19-positive DTCs in BM after surgery in their study [10]. Thus, the change in the relative contribution of the three markers before and after surgery observed in our study is an interesting finding, and may suggest a differential marker expression in the DTCs detected at various sampling time points. Whether this reflects a general change in the expression profile of the DTC population present in the BM before and after surgery, may be a topic of future investigation.

Despite the establishment of the prognostic and predictive significance of BM DTCs [2,16,19], detection of DTCs is yet to be adapted in clinical routine staging procedures. One of the reasons is the challenge of standardization of methods. This has been addressed for immunocytochemical methods [22], but not in a similar way for RT-PCR methods. Additionally, patient discomfort and logistical challenges involved with BM sampling might be a hurdle to routine use. The rapid development of methods to detect circulating tumor cells (CTCs) in peripheral blood [23-25], may offer a solution to this problem if CTCs are demonstrated to be of equivalent relevance to clinical outcome. However, in non-metastatic breast cancer patients only a limited number of studies have so far compared BM and peripheral blood examinations directly, by sampling both blood and BM at the same time point from the patients [26-28]. Based on these studies the clinical significance of CTCs in peripheral blood seems less clear than for DTCs in BM in this patient group (reviewed in [29]). Nevertheless, the clinical utility of the prognostic information from DTCs will also depend on the development of treatment options specifically targeting DTCs and CTCs. Detection and isolation techniques that allow a molecular characterization of DTCs may provide tools to guide novel, targeted therapies [29]. However, presently ASCO guidelines state that the data from DTC detection in BM and CTC detection in blood, even in metastatic breast cancer patients, are insufficient to recommend assessment of minimal residual disease for the management of patients with breast cancer [30]. Further validation in randomized trials is needed to confirm the clinical value of minimal residual disease detection.

## Conclusion

Detection of DTCs by our MM mRNA panel in BM collected after surgery identified non-metastatic breast cancer patients at high risk for systemic relapse, and with reduced breast-cancer specific survival. Furthermore, patients with positive DTC status both before and after surgery are recognized as patients with an extremely poor prognosis. Thus, a strategy involving a BM aspirate during general anesthesia, prior to surgery, with a repeated, post-operative aspiration performed only among patients who were DTC positive in the first sample, should be feasible and having significant clinical relevance. Moreover, although we may not conclude on the potential of DTC status for monitoring the efficiency of adjuvant treatment, the strong prognostic effect of DTCs observed after surgery suggests that such monitoring may be of importance. Future studies are required to determine whether the patients with inferior outcome predicted by DTC detection, especially those with DTCs detected both before and after surgery, may benefit from intensified or secondary adjuvant therapy.

## Competing interests

The authors declare that they have no competing interests.

## Authors’ contributions

KT carried out all the qRT-PCR analyses, performed the statistical analyses and drafted the manuscript. SO did all the RNA purification, and participated in manuscript preparation. RH participated in sample collection, study design and manuscript preparation. Statistician JTK participated in the statistical evaluation, and preparation of the manuscript. BG participated in sample collection, and manuscript preparation. JMR gave scientific advice, contributed to data interpretation and manuscript preparation in addition to correcting the English grammar. RS is the group leader and contributed to result interpretation, supervision and manuscript preparation. ON participated in the study design, coordinated the study, gave statistical advice and contributed to the manuscript preparation. All authors read and approved the final manuscript.

## Pre-publication history

The pre-publication history for this paper can be accessed here:

http://www.biomedcentral.com/1471-2407/12/190/prepub
